# Soluble triggering receptor expressed on myeloid cells as a marker of non-infectious systemic inflammatory response syndrome

**DOI:** 10.1186/cc9840

**Published:** 2011-03-11

**Authors:** E Grigoryev, A Golovkin, V Matveeva, G Plotnikov, D Shukevich

**Affiliations:** 1 Institute of Complex Problems of Cardiovascular Diseases, Kemerovo, Russia

## Introduction

The objective was to determine the diagnostic significance of soluble triggering receptor expressed on myeloid cells (sTREM-1) as a marker of the systemic inflammatory response syndrome (SIRS) in ischemia/reperfusion (extracorporeal circulation).

## Methods

Eighty-nine patients were included in the study. All patients were divided into: group 1 (*n *= 41) - coronary heart disease (CHD), group 2 (*n *= 47) - acquired heart diseases (AHD). All the operations were performed with normothermal nonpulsatile extracorporeal circulation (EC) with cold blood cardioplegia (coronary artery bypass surgery in the group with CHD and prosthetics/plastic valves for the group with AHD). Systemic inflammatory response (SIRS) was defined by Bone and colleagues [1]; ischemia and reperfusion by lactate and oxygen status of arterial and mixed venous blood (StatProfile). We studied by enzyme immunoassay level (ELISA): high-sensitivity C-reactive protein (hsCRP), procalcitonin (PCT-Q) and sTREM-1, using the sets by Bender Medsystems, CanAg and Brahms PCT-Q. Data are presented as mean ± standard deviation.

## Results

All patients registered the increased level of hsCRP, without significant difference between the two groups. At the point after the operation, the rate of hsCRP was significantly higher for the group AHD. Correlations were noted between levels of hsCRP and the frequency of occurrence of criteria for SIRS (*r *= 0.22 for the group of IHD, *P *= 0.03; *r *= 0.39 for the group AHD, *P *= 0.01). The odds ratio (OR) likelihood of SIRS complications on hsCRP was 2.4 in the group with CHD and 3.9 in the group with AHD. There was no significant difference between the rates of PCT for the corresponding points of comparison groups. The highest predictive value (OR = 2.9, *P *= 0.03) has a PCT in relation to the severity of SIRS in patients with AHD (infectious endocarditis and rheumatic heart disease). The sTREM-1 level was higher compared with the postoperative period (55.5 ± 8.8 vs. 77.8 ± 9.1 pg/ml, *P *= 0.005; 49.9 ± 6.7 vs. 87.5 ± 8.9 pg/ml, *P *= 0.004). We studied the correlation between the level of sTREM-1 and the frequency of occurrence of symptoms SIRS (*r *= 0.77 for the group of IHD, *P *= 0.002; *r *= 0.79 for the group AHD, *P *= 0.04). The OR sTREM-1 probability of SIRS complications was highest in comparison with all of the markers.

## Conclusions

sTREM-1 has the greatest diagnostic significance in relation to non-infectious SIRS in ischemia/reperfusion.
